# Risk Factors Associated with Postnatal Growth Velocity in Preterm Infants in a Tertiary Hospital in Northeast Mexico

**DOI:** 10.3390/jcm14238489

**Published:** 2025-11-30

**Authors:** Esteban López-Garrido, Sergio Alberto Márquez-Moreno, Araní Casillas-Ramírez, Rodrigo Vargas-Ruiz, Elsa Verónica Herrera-Mayorga, Hadassa Yuef Martínez-Padrón

**Affiliations:** 1 Unidad de Cuidados Intensivos en Neonatos, Hospital Regional de Alta Especialidad Ciudad Victoria, Servicios de Salud del Instituto Mexicano del Seguro Social Para el Bienestar (IMSS-BIENESTAR), Libramiento Guadalupe Victoria S/N, Área de Pajaritos, Victoria 87087, Tamaulipas, Mexico; estebanlopez_garrido@hotmail.com; 2 Facultad de Medicina, Universidad Autónoma de Tamaulipas, Matamoros 87300, Tamaulipas, Mexico; sergioalbertomarquezmoreno@hotmail.com; 3 Hospital Regional de Alta Especialidad Ciudad Victoria, Servicios de Salud del Instituto Mexicano del Seguro Social Para el Bienestar (IMSS-BIENESTAR), Libramiento Guadalupe Victoria S/N, Área de Pajaritos, Victoria 87087, Tamaulipas, Mexico; 4 Subdirección de Enseñanza e Investigación, Hospital Regional de Alta Especialidad Ciudad Victoria, Servicios de Salud del Instituto Mexicano del Seguro Social Para el Bienestar (IMSS-BIENESTAR), Libramiento Guadalupe Victoria S/N, Área de Pajaritos, Victoria 87087, Tamaulipas, Mexico; acasillas.hraev@gmail.com; 5 Unidad Académica Multidisciplinaria Mante, Universidad Autónoma de Tamaulipas, Mante 89800, Tamaulipas, Mexico; rodvargas@uat.edu.mx (R.V.-R.); evherrera@docentes.uat.edu.mx (E.V.H.-M.)

**Keywords:** preterm, growth velocity, length, head circumference, associated factors

## Abstract

**Background**: Suboptimal growth velocity (GV)—weight < 15 g/kg/day, head circumference (HC) < 0.9 cm/week, and length < 1 cm/week—are related to neurodevelopmental problems. Comorbidities such as sepsis, patent ductus arteriosus, bronchopulmonary dysplasia, hemodynamic instability with use of inotropes, and necrotizing enterocolitis, among others, have been related to suboptimal GV. Therefore, this study aimed to evaluate GV in preterm newborns in the Neonatal Intensive Care Unit of a tertiary-level hospital in Mexico in the first 28 days of life and determine the main factors related to suboptimal GV. **Methods**: This was an observational, descriptive, retrospective, and longitudinal study. Thirty-one PNBs admitted to the NICU from March 2021 to February 2023 were included. Descriptive statistics were used for neonatal characteristics and factors. They were classified into group 1 (GV < 15 g/kg/day) and group 2 (>15 g/kg/day) and compared with Fisher’s exact test. Multivariate linear regression analysis was performed for factors related to suboptimal GV. **Results**: Among 31 preterm infants (mean GA 30 ± 3 weeks; birth weight 1241 ± 377 g), the mean growth velocity (GV) at 28 days was 16.5 ± 10 g/kg/day for weight (optimal), while length (0.7 ± 0.4 cm/week) and head circumference (0.41 ± 0.28 cm/week) remained suboptimal. Overall, 42% showed GV < 15 g/kg/day, and 58% achieved optimal GV. Multivariate analysis revealed that apnea of prematurity (β = –9.8; *p* = 0.023) and birth weight < 1000 g (β = –6.3; *p* = 0.026) were negatively associated with GV, whereas mixed feeding (breast milk + formula) had a positive effect (β = +6.3; *p* = 0.008). **Conclusions**: The 31 PNBs studied had a mean GV of optimal weight, and a mean GV of length and HC suboptimal to that suggested by the guidelines. The degree of low birth weight (LBW) and apnea of prematurity had a negative relationship with GV, while the type of feeding showed a positive relationship (*p* = 0.026, 0.023, and 0.008, respectively).

## 1. Introduction

Preterm birth continues to be a global health problem. According to the World Health Organization (WHO), a baby born alive before 37 weeks of gestation is considered preterm. Preterm infants are divided into subcategories according to gestational age: extreme preterm (less than 28 weeks), very preterm (28 to 32 weeks), and moderate-to-late preterm (32 to 37 weeks). There is also a category according to birth weight, classifying them into low birth weight (LBW, less than 2500 g at birth), very-low birth weight (VLBW, less than 1500 g at birth), and extremely low birth weight (ELBW, less than 1000 g at birth) [1,2]. Growth is the most sensitive index of health and a fundamental parameter for nutritional assessment. An adequate growth pattern in the first years of life is essential for ensuring normal neurosensory development. Growth assessment should be performed by weight, length, and head circumference (HC), up to 24 months of age, using the gestational age (GA) corrected up to 2 years for all the referred parameters [3]. After birth, there is a weight loss and a delay in GV in PNBs with respect to fetuses of the same gestation age, with recovery of birth weight between 10 and 30 days later. Premature newborns that are more premature and have a lower birth weight have a higher body water content, a lower amount of lean mass, and a greater insensible water loss. Therefore, they are prone to greater weight loss in the first week of life and greater difficulty in regaining weight, especially if their condition is serious (Zamorano-Jiménez C. A., 2012) [4]. Subsequently, growth accelerates, but the loss is not fully recovered [5].

Moreover, adequate nutrition in the newborn (NB) is strongly related to lower morbidity and mortality and to a better neurodevelopmental prognosis in the medium and long term [6]. Malnutrition and deficiency in nutritional intake are common in hospitalized infants due to decreased nutrient intake, prolonged fasting, variation in requirements in different stressful situations, and disorders in nutrient metabolism [7].

The WHO recommends the use of the Fenton growth chart curves for preterm neonates up to 50 weeks of gestation, which include weight, length, and head circumference indicators [8,9]. The American Academy of Pediatrics (AAP) recommends that the goal of nutrition should be to achieve adequate postnatal growth at GA, defined not only by anthropometric parameters (15 g/kg/d or 20 to 30 g/kg/d; 1 cm/week of length and HC) but also by the variation in body composition and retention of different nutrients [10].

In this context, Zamorano-Jiménez C.A., et al. (2012), in a descriptive, retrospective study of 101 newborns under 1500 g, observed a maximum percentage of weight loss of 8.6 ± 4.5%; weight recovery occurred on day 10.9 ± 5.2, with the greatest weight loss in those with the lowest GA [4]. Additionally, Lango M. O. et. al., in 2013, reported a GV of 14 g/kg/day in a retrospective cohort of 51 NBs with extremely low birth weight (ELBW); this figure is similar to that suggested in the guidelines, but no significant association between GV and type of feeding, days to complete total enteral feeding, time to regain birth weight, intraventricular hemorrhage (IVH), intrauterine growth restriction (IUGR), or use of prenatal steroids was found [11]. Moreover, Hair, et al. (2013) [12] evaluated GV in PNBs under 1250 g that were exclusively breastfed. The postnatal growth restriction up to 34 SDG was corrected by fortification at 60 mL/kg/d in a single-center prospective cohort of 104 NBs with a mean of 27.6 weeks and weight of 913 g, observing a weight gain of 24 g/kg/d, a height gain of 0.99 cm/week, HC gain of 0.72 cm/week. [12].

On the other hand, Espinosa Reyes et al., in 2013 [13], conducted a prospective descriptive study of preterm newborns (PNBs). The sample consisted of 73 patients, 37 of whom were female (50.7%). A total of 60.2% were born between 32 and 36 weeks of gestation, 50.6% weighed between 1200 and 1399 g, and 36% measured between 38 and 40.9 cm. At 3 months, 70% were below the 3rd percentile for height/age and weight/age, and at 1 year, more than 50% had reached normal percentiles. A lower gestational age, singleton pregnancy, exclusive breastfeeding (68.5%), longer duration of breastfeeding, and the absence of infections were associated with statistically significant weight gain [13]. Also, in a study conducted in Spain by García-Muñoz, et al. in 2017 that included 4520 extremely PNBs, the rate of weight gain was below that suggested by the guidelines (birth–28 days 8 g/kg/d, 28 days–36 weeks 14.3 g/kg/d, and from 36 weeks corrected to hospital discharge 11.4 g/kg/d), with postnatal restriction affecting height and weight to a greater degree [14].

In a prospective cohort of 144 PNBs, Raturi S., in 2017, observed that their GV was lower than that recommended by the AAP (15 g/kg/day) at 28 days (9.8 g/kg/day) and at discharge (11.8 g/kg/day). The use of inotropic drugs, bronchopulmonary dysplasia, and necrotizing enterocolitis (NEC) were factors thought to have had negative effects on GV at discharge; also, in patients with patent ductus arteriosus (PDA), NEC, and sepsis, enteral feeding was delayed and required more time was require to obtain complete enteral feeding. In contrast, there was a positive association between GV and water, protein, and energy intake, at discharge [15]. Lastly, Khan Z., et al., in 2018 [16], studied a prospective cohort of very preterm and extremely preterm infants. After the implementation of a standardized feeding protocol kept both groups below their weight-for-age, the authors found no significant difference in growth until the fifth week, but did find a significant difference at discharge, where the very preterm infants showed better weight-for-age positioning and shorter NICU stays. The GVs in HC and length were higher in very preterm infants compared to extremely preterm infants [16].

Despite existing evidence linking suboptimal growth velocity to adverse neurodevelopmental outcomes and various comorbidities, there remains a lack of comprehensive data on the specific factors influencing growth velocity during the critical first 28 days of life in preterm newborns within our regional NICU context. This study, therefore, fills this gap by providing a focused evaluation of growth velocity and its associated factors in this vulnerable population, offering insights relevant for optimizing neonatal care in similar settings. Thus, the present study aimed to evaluate GV in preterm newborns (PNBs) in the Neonatal Intensive Care Unit (NICU) of a tertiary level hospital in northeast Mexico in the first 28 days of life and determine the main factors related to suboptimal GV.

## 2. Materials and Methods

Study design

Observational, descriptive, retrospective, and longitudinal.

Location and site of the study

Neonatal Intensive Care Unit and Neonatology Service of the Hospital Regional de Alta Especialidad Ciudad Victoria, Servicios de Salud del Instituto Mexicano del Seguro Social para el Bienestar (IMSS-BIENESTAR).

Sample size

We included records of patients admitted to the NICU with a diagnosis of prematurity (less than 37 SDG at birth) who underwent weekly anthropometric measurements from the date of admission until 28 days of extrauterine life or the date of discharge at the Regional High Specialty Hospital of Ciudad Victoria “Bicentenario 2010” during the period from 1 March 2021 to 28 February 2023, and who met the selection criteria.

Inclusion criteria:

- Records of patients who had information regarding their main anthropometric measurements (weight, height, and WC) during their stay in the NICU service up to 28 days or their discharge.
- Records of patients who met the WHO definition of “prematurity”.

Exclusion criteria:

- Incomplete records or a lack of anthropometric records that interfered with the performing this study.
- Records of newborns with genetic defects or malformations incompatible with life.
- Records of newborns with congenital metabolic defects.
- Records of NBs who died during their stay in the NICU.

Data retrieval and analysis

Patient records were collected for patients who met the inclusion criteria at the Hospital Regional de Alta Especialidad de Ciudad Victoria—IMSS-Bienestar (HRAEV) during the period from 1 March 2021, to 28 February 2023.

The electronic and physical patient records of the patients were obtained from the MedSys system (Medical Information System) v6.3.1 of the unit to obtain sociodemographic, prenatal, perinatal, and hospital stay data, such as nutritional practices, morbidities that could affect GV, such as sepsis, NEC, respiratory distress syndrome (RDS), patent ductus arteriosus (PDA), and management, such as the use of parenteral nutrition (PN), amines, and mechanical ventilation in the NICU of the HRAEV.

The following formula was used to evaluate the weekly GV in patients who met the inclusion criteria for this study:

$$GV=\frac{PWg-CWg}{d}\times \frac{PWk+CWk}{2}$$

where GV is the growth rate, PWg is the previous weight in grams with respect to the last measurement at the time of the new measurement, CWg is the current weight in grams at the time of the last measurement taken for the date, d is the number of days difference between both measurements, PWk is the previous weight in kilograms with respect to the last measurement at the time of the new measurement, and CWk is the current weight in kilograms at the time of the last measurement taken for the date.

After collecting these data, they were evaluated together with the associated comorbidities in each of the patients and, in this way, were used to determine whether there were associated factors that modified this somatometric parameter, either increasing or decreasing its total value.

Incubators and Feeding Protocol in NICU PNBs are cared for in radiant heat incubators, specifically the Dräger Babytherm^®^ 8004/8010 model (Drägerwerk AG & Co. KGaA Moislinger Allee 53–55 23558 Lubeck, Germany), set to a target temperature of 36.8 °C with automatic temperature regulation. The incubators are operated without added humidity. To minimize fluid loss in the patients, a low-density linear polyethylene plastic cover (gauge 50, manufactured by Triplast, Querétaro, Querétaro, México) is applied. 

In the unit, fluid management in preterm neonates are individualized according to birth weight. For infants weighing less than 800 g, fluid administration is initiated at 100 mL/kg/day; for those under 1000 g, fluid administration is initiated at 90 mL/kg/day; for neonates weighing less than 1500 g, fluid administration is initiated at 80 mL/kg/day; and for those over 1500 g, fluid administration is initiated at 70 mL/kg/day. Subsequent intravenous fluid increments are adjusted dynamically based on the calculation of insensible water losses using the formula fluid intake minus measurable outputs plus weight loss or reduced weight gain equals insensible losses. The calculation accounts for an expected percentage of weight loss according to birth weight categories (<800 g: 15–20%, 800–1000 g: 12–15%, 1000–1500 g: 10–12%, >1500 g: 5–10%), using an ideal (expected) weight as a reference. This approach allows for tailored fluid management adapted to the individual physiological needs of each patient.

The enteral nutrition protocol followed in the Neonatal Intensive Care Unit of the HRAEV is based on established methodologies and adapted to local patient needs. Enteral feeding volumes are initiated according to birth weight: 12.5 mL for neonates weighing less than 1000 g, 20 mL for those around 1000 g, and 25 mL for neonates above 1500 g. Fluid management starts from the first day of life, with administration volumes as previously described by weight categories and subsequently adjusted according to fluid balance calculations based on the following equation:

Measurable intake (intravenous fluids, medications) − quantifiable output (urine, feces, orogastric tube losses) + weight loss or − weight gain = Insensible Losses

In the Neonatal Intensive Care Unit at HRAEV, the nutrition protocol follows the guidelines established by the European Society for Paediatric Gastroenterology, Hepatology and Nutrition (ESPGHAN). Protein intake is initiated at 2 g per kilogram per day, with daily increments of 0.5 g up to a maximum of 3.5 g. Lipid provision starts at 1 g per kilogram per day, increasing daily by 0.5 g to a maximum of 3 g. Glucose is administered at 4 to 6 mg/kg/min, with daily increments of 2 mg, reaching a maximum range of 10 to 12 mg/kg/min.

Every PNB in the NICU used the following feeding protocol based on the Cochrane neonatal meta-analysis, adapted to our local context. During the first week of life, colostrum therapy combined with trophic enteral stimulation is implemented by administering 0.1 mL of colostrum evenly distributed across buccal mucosae every 4 h, especially for neonates with ELBW at birth.

Trophic enteral feeding is performed using a fortifier (Enfamil^®^ Human Milk Fortifier MEAD JOHNSON & COMPANY, LLC. 2400 W. Lloyd Expressway, Evansville, IN, USA) for ELBW neonates. A total of 1 mL via orogastric tube is given every 8 h during the initial 5 days, while neonates weighing between 1000 and 1250 g receive 12.5 mL/kg/day for 3 days, as clinical conditions permit. For neonates weighing 1250 to 1500 g, feeding is initiated at 15 to 20 mL/kg/day, and for those above 1500 g, feeding is initiated at 20 to 30 mL/kg/day. Alimentation occurs every 2 h for PNBs weighing <750–1250 g and every 3 h for PNBs weighing 1251→2500 g. Additionally, for PNBs with an indication of PN, a formula of 24 kcal/oz was used.

When exclusively breastfed, fortification is achieved using a fortifier (Enfamil^®^ Human Milk Fortifier) to achieve a daily intake of 100 mL/kg/day. If a fortifier is unavailable, a mixed diet of breast milk and formula (PreNan^®^ 24 cal/oz, manufacturer by Nestlé México, Ciudad de México, México) is used.

Volume increments are stratified according to birth weight. Infants < 750 g receive increments of 12.5 mL/kg/day; those 1000 to 1250 g receive increments ranging from 12.5 to 15 mL/kg/day; neonates weighing 1250 to 1500 g receive 15 to 20 mL/kg/day; and those >1500 g receive 20 to 30 mL/kg/day.

Statistical analysis

Descriptive statistics were used for continuous variables by calculating means and standard deviations. Student’s t-tests were used to compare patients who were discharged with normal weight, height, and HC or with low weight, height, and HC. Frequencies and percentages were calculated for categorical variables and compared using Fisher’s exact test. To determine the association between independent variables and growth velocity, a multivariate linear regression model was fitted. Values of *p* < 0.05 were considered statistically significant. SPSS 22 software was used for statistical analysis.

## 3. Results

In the study period, which was from 1 March 2021, to 28 February 2023, a total of 156 newborns were admitted to the NICU of the HRAEV, of which 91 (58%) were premature; 60 files were excluded because they did not meet the inclusion criteria: 50 had incomplete anthropometric measurements, 8 records corresponded to newborns with genetic defects or malformations, and 2 records corresponded to PNBs with metabolic defects. The sample consisted of 31 files, which were reviewed and analyzed: 19 (61%) were female and 12 (39%) male; with a mean GA of 30 ± 3 weeks; mean birth weight of 1241.7 ± 377.4 g; mean length of 37.2 ± 3.6 cm; mean HC of 26.5 ± 2.7 cm; and mean Miller’s index of 1.39 ± 0.06. In terms of prematurity grade grouping, 6 (19%) were less than 28 weeks of gestational age, 16 (52%) were from 28 to 32 weeks of gestational age, and 9 (29%) were older than 32 of weeks gestational age. In terms of LBW grouping, 8 (26%) weighed less than 1000 g, 16 (52%) weighed from 1000 to 1500 g, and 7 (22%) weighed more than 1500 g. In terms of to their weight for GA: 26 (84%) were adequate and 5 (16%) were underweight; 23 (74%) were singleton pregnancy products and 8 (26%) were multiple pregnancy products; 30 (97%) were delivered by cesarean section and 1 (3%) vaginally; 15 (48%) received prenatal steroids; 19 (61%) had invasive mechanical ventilation; 11 (35%) had aminergic support. In relation to the type of feeding: 7 (23%) received breast milk, 6 (19%) received formula milk and 18 (58%) received mixed feeding. The day of onset of PN had a mean of 2.2 + 0.6. Regarding the neonatal factors analyzed, 5 (16%) had sepsis, 5 (16%) NEC, 3 (10%) HIV, 19 (61%) RDS, 7 (23%) DAP, 12 (39%) presented with apneas, and 28 (90%) received antibiotics.

The average weight loss in the first week was 10.9 + 6%. The mean GV at 28 days of life was 16.5 + 10 g/kg/day for weight, 0.7 + 0.4 cm/week for length, and 0.41 + 0.28 cm/week for HC. They were classified into two groups according to GV at 28 days of life: group 1: <15 g/kg/day, consisting of 13 patients, and group 2: >15 g/kg/day, consisting of 18 patients. Regarding height and HC, all showed growth below what is considered adequate at 28 days of life (<1 and 0.9 cm/week, respectively). Table 1 shows the sociodemographic data and neonatal factors of the patients evaluated.

The 28–32 weeks gestation and 1000–1500 g birth weight groups showed better GV, with a significant difference in weight gain. No statistically significant difference was found when comparing the other neonatal characteristics and factors in both groups. A difference was only observed when comparing sex (Table 2).

Nutritional intake in the first week of life was also analyzed. We investigated if there were any significant differences in relation to carbohydrate, amino acids, lipids, and caloric intake between the groups with GV < 15 g/kg/day and >15 g/kg/d (Table 3).

At 28 days of life, the mean percentile for height and HC was above the 10th percentile, but with a wide standard deviation; however, the mean percentile for weight was below the 10th percentile. When comparing the percentiles at 28 days of life between the two groups (group 1 and group 2), it can be observed that those with an optimal GV (>15 g/kg/day) reached a mean percentile above the 10th percentile in weight, height, and HC. For the classification of the newborns according to weight for gestational age, we use Fenton tables, considering the newborn’s weight adequate for gestational age when it is between the 10th and 90th percentile, low when it is below the 10th percentile, and high when it is above the 90th percentile. When comparing the percentiles at 28 days between group 1 and group 2, no statistically significant difference was found (weight *p* = 0.237, height *p* = 0.718 and PC *p* = 0.981) (Table 4).

Through a multivariate linear regression model, the association of neonatal characteristics and factors was estimated to predict the effect of the LBW group, feeding type, and apnea of prematurity on weight GV (g/kg/d) at 28 days of life; the regression equation was statistically significant, F 5.9, *p* 0.023, β −0.46. The R-squared value was 0.17, indicating that 17% of the change in GV of g/kg/d can be explained by the model using the variables apnea, type of feeding, and degree of low birth weight. The regression equation was 30.5 + 9.8 for each patient presenting apnea, +4.6 according to feeding type, and +6.3 according to LBW grade, where feeding type increases by 6.3 g/kg/d each time a patient receives mixed feeding and the presence of apnea and a birth weight below 1000 g decreases it by 9.8 and 6.3 g/kg/d, respectively.

## 4. Discussion

Growth monitoring of weight, height, and HC in neonates is a widely used way of assessing GV to compare it with what is suggested by national and international guidelines as optimal and, in this way, make pertinent interventions to reduce the repercussions on neurodevelopment in the medium and long terms. This underscores the clinical importance of timely and accurate growth assessment to guide individualized nutritional and medical management in preterm infants. The objective of this study was to evaluate the GV of preterm infants at 28 days of life, since we consider that this is the period in which the neonate has often stabilized and managed to establish its growth pattern. At the same time, we had the intention of exploring the different neonatal factors that could have a positive or negative effect, especially on weight gain. In this study, we found a decrease in weight in the early postnatal period that was most likely associated with water loss, which may be exacerbated by the lack of moisture management and heating, in addition to the fact that they are in a highly catabolic stage. Weight loss in the first week of life was greater in the group with adequate GV compared to those with suboptimal GV, which seems to be related to the fact that the group with adequate GV was composed of a greater proportion of infants < 32 weeks of gestational age at birth (83% vs. 53%). The overall mean weight gain at 28 days of life was adequate as suggested by the guidelines; however, this was not the case for height and HC. When comparing the groups with optimal and suboptimal weight gain, only sex showed a significant difference in both groups, with no difference in the remaining variables. These findings suggest the need to monitor multiple growth parameters beyond weight alone to fully understand growth patterns.

Previously, Martin, et al. (2009) [5] evaluated GV at 28 weeks of age in preterm infants, finding that nutritional practices (volume, type, and composition of PN, as well as type and volume of enteral feeding) in the first week of life showed a positive effect on GV. In our study, mixed enteral feeding (breast milk plus formula) showed a positive effect on GV [5]. Also, Zamorano-Jiménez, et al. (2012) reported a weight loss in the first week of life lower than that our study (8.6% vs. 10.9%), which is probably associated with the management of the thermal environment and humidity; however, the GV was similar to that reported in our study [4]. Additionally, in a study conducted in South Africa by Lango, et al. (2013) [11] in a retrospective cohort of extremely preterm infants, a GV slightly below that reported in our study for the same patient population (14 vs. 16 g/kg/d) was reported; however, although it was not statistically significant, when comparing the < 1000 g vs. 1000–1500 g group, we did observed some difference between them (16 vs. 17.9 g/kg/day). On the other hand, the previous study did not find a statistically significant association between the type of feeding, time to reach full enteral feeding, HIV, steroid exposure, or weight recovery time, most likely associated with the size of the population studied [11]. These comparisons highlight variations across populations and underscore the complexity of factors influencing growth velocity, suggesting the need for larger multicenter studies.

Contrastingly, Hair, et al. (2013) [12] reported adequate growth and a low incidence of extrauterine growth restriction in preterm infants who were less than 1250 g and exclusively fed with breast milk. In our study, we found a positive relationship with mixed feeding, possibly because in recent years we have not had human milk fortifiers; milk fortifiers are included standardized early fortification feeding protocols [12].

Unlike Espinosa Reyes, in our study we found no positive impact on weight gain with exclusive breastfeeding, absence of infections, and lower gestational age; on the other hand, he also reported that 70% of preterm infants showed growth restriction at 3 months, with recovery of 50% by the first year of life [13].

Likewise, García-Muñoz, et al. (2017) [14] authored a cohort study of 4522 white RNs with a GA ≤ 28 weeks, reporting a GV at 28 days of life of 8 g/kg/d, which is much lower than what we reported in our study. This is most likely associated with the fact that his population included immature preterm infants younger than 25 weeks, unlike our study where the minimum GA was 25 weeks. Nevertheless, similar to our study, despite the difference in populations, GV in the first weeks is low [14].

In contrast to Khan Z, who in his cohort study found that extremely preterm infants (<28 weeks) presented a lower growth pattern in weight, length, and HC compared to those >28 weeks (probably associated with having a later cumulative intake of proteins, fats and carbohydrates), we did not find this relationship in our study due to the sample size [16].

In the national context, the GV of length and HC was found to be lower in our study compared to that reported by Mercado Aviles L. in a Mexican study performed in preterm infants in 2018 (0.7 and 0.4 cm/week vs. 0.86 and 0.47 cm/week) [17]. Also, similar to Acevedo-Ortiz, et al. (2018) [18], we observed weight loss in the first week in the study group and improvement in weight gain after the second week of life. In our study, we did not observe any effect of low weight for gestational age at birth, unlike Acevedo-Olguin, who reported that there was a reduction in GV at week 8, with an RR of 4.4 for presenting low weight at discharge [18].

Finally, in our study, the factor with the strongest relationship to suboptimal GV was the presence of apneas. Apnea is defined as the cessation of breathing for 20 s or more, or less than 20 s when accompanied by bradycardia (less than 100 beats per minute) or oxygen desaturation (less than 90% oxygen). Frequent apnea is defined as apnea episodes that occur six or more times per hour, and severe apnea is defined as apnea requiring positive pressure ventilation with a bag and mask for recovery. Patients who required gentle tactile stimulation and whose apnea was not considered frequent or severe were not included in the study. The apnea, which may be associated with difficulty in achieving early full enteral feeding, increased caloric intake due to stress events produced by the apnea and reduced growth hormone release due to interrupted periods of sleep and peripheral resistance to insulin-like growth factors (IGF) and IGF-binding protein 3. These factors have been mostly studied in the pediatric population and shown to increase after the correction of sleep apnea, followed by improvement in growth patterns [19,20]. It is worth mentioning that so far there are no articles that have evaluated the relationship between apnea of prematurity and GV. However, this study is limited by the small sample size and its retrospective design, which reduce the generalizability of the findings. Future prospective studies with larger cohorts are needed to confirm these associations and to explore the mechanisms underlying the impact of apnea on growth velocity. Understanding these practical implications is essential for adequately guiding clinical interventions. Early management of apnea, including both its therapeutic approach and the evaluation of possible central etiologies, is a determining factor in the patient’s clinical outcome. Furthermore, breast milk represents the strategy of choice for promoting tolerance to enteral feeding in premature newborns, while its fortification is essential to ensure adequate weight gain and optimal development.

## 5. Conclusions

The GV, expressed in g/kg/d (weight), in the group of patients studied presented an optimal mean at 28 days of life according to the AAP guidelines. The GV in cm/week (height and WC) was below the optimum according to the AAP guidelines. There is no relationship between the degree of prematurity and GV at 28 days of life in our study population. LBW (<1000 g) and the presence of apneas showed a negative relationship with GV at 28 days of life, while mixed feeding showed a positive relationship. The nutritional components (carbohydrate, amino acid, lipid, and caloric intake) in the first week of life had no significant effect on GV.

## Figures and Tables

**Table 1:** Neonatal characteristics and factors of the patients studied.

Neonatal Characteristics and Factors	Total n: 31 (100%)	Group 1 (<15 g/kg/d) n: 13 (42%)	Group 2 (>15 g/kg/d) n:18 (58%)	*p*
Sex				
Female	19 (61)	11 (84.6)	8 (44.4)	0.032
Male	12 (39)	2 (15.4)	10 (55.6)	
Gestational age	30 ± 3	30.9 ± 3.2	29.6 ± 2.8	0.215
Birth weight (g)	1241 ± 377	1276 ± 341	1216 ± 409	0.671
Size (cm)	37 ± 3.6	37.5 ± 3.6	37 + 3.7	0.727
Head circumference (cm)	26.5 ± 2.7	26.8 ± 2.6	26.4 + 2.7	0.711
Miller Index	1.39 ± 0.06	1.39 ± 0.07	1.39 + 0.06	0.990
Gestational age group				
<28 weeks	6 (19)	2 (15.4)	4 (22.2)	0.203
28–32 weeks	16 (52)	5 (38.4)	11 (61.1)	
>32 weeks	9 (29)	6 (46.2)	3 (16.7)	
Birth weight group				
<1000 g	8 (26)	3 (23)	5 (28)	0.651
1000–1500 g	16 (52)	6 (46)	10 (55)	
>1500 g	7 (22)	4 (31)	3 (17)	
Weight for gestational age				
Under			3 (17)	1.00
Suitable	5 (16)	2 (15)	15 (83)	
Pregnancy	26 (84)	11 (85)		
Only	23 (74)	10 (77)	13 (72)	1.00
Multiple	8 (26)	3 (23)	5 (28)	
Birth pathway				
Vaginal	1 (3)	1 (8)	0 (0)	0.419
Cesarean section	30 (97)	12 (92)	18 (100)	
Prenatal steroids	15 (48)	7 (54)	8 (44)	0.605
Invasive mechanical ventilation	19 (61)	8 (61)	11 (61)	0.981
Use of amines	11(35)	4 (31)	7 (39)	0.718
Type of power supply				
Breast milk	7 (23)	3 (23)	4 (22)	0.891
Formula milk	6 (19)	3 (23)	3 (17)	
Mixed	18 (58)	7 (54)	11 (61)	
Start day NP	2.2 ± 0.6	2.4+ 0.7	2.1+ 0.3	0.111
Sepsis	5 (16)	3 (23)	2 (11)	0.625
Necrotizing enterocolitis	5 (16)	2 (15)	3 (17)	1.00
Intraventricular hemorrhage	3 (10)	1 (8)	2 (11)	1.00
RDS	19 (61)	8 (61)	11 (61)	0.981
Patent ductus arteriosus	7 (23)	3 (23)	4 (22)	1.00
Apnea	12 (39)	3 (23)	9 50)	0.158
Antibiotics	28(90)	12 (92)	16 (89)	1.000
Weight loss in the first week (%)	10.9 ± 6	9.7 ± 5.8	11.8 ± 6.3	0.345
Weight g/kg/day at 28 days of life	16.5 ± 10	7.2 + 5.2	23.2 ± 7.7	0.000
Size cm/week at 28 days of age	0.70 ± 0.44	0.73 ± 0.38	0.67 ± 0.49	0.690
HC cm/week at 28 days of life	0.41 ± 0.28	0.40 ± 0.24	0.41 ± 0.32	0.957

PN: parenteral nutrition, RDS: respiratory distress syndrome, HC: head circumference.

**Table 2:** Comparison of growth velocity by weight groups and weeks of gestation.

By Gestational Age at Birth					
Parameter	<28 weeks	28–32 weeks	>32 weeks	*p*	CI
Weight (g/kg/d)	16.9 + 15	18 + 9.4	13.5 + 8.9	0.007	10–21
Size (cm/week)	0.29 + 0.34	0.85 + 0.44	0.69 + 0.35	0.067	−0.10–0.3
HC (cm/week)	0.25 + 0.22	0.48 + 0.32	0.38 + 0.22	0.031	0.08–0.65
**By birth weight group**					
Parameter	<1000 g	1000–1500 g	>1500 g	*p*	CI
Weight (g/kg/d)	16.0 + 13	17.9 + 9.5	14.0 + 9.8	0.005	11–20
Size (cm/week)	0.32 + 0.31	0.93 + 0.41	0.58 + 0.33	0.075	−0.15–1.3
HC (cm/week)	0.29 + 0.21	0.53 + 0.32	0.26 + 0.13	0.052	−0.007–0.72

HC: Head circumference; CI: Confidence interval.

**Table 3:** Nutritional and caloric intake during the first week of life.

Nutritional/Caloric Contribution	Total	Group 1	Group 2	*p*
Carbohydrates g/kg/day	13.4 ± 3.1	13.1 ± 2.9	13.5 ± 3.3	0.755
Amino acids g/kg/day	3.2 ± 0.9	3.4 ± 0.5	3.1 ± 1.1	0.348
Lipids g/kg/day	2.6 ± 0.7	2.7 ± 0.5	2.5 ± 0.9	0.546
Calories	89.2 ± 19.3	91 ± 16.5	88 ± 21.4	0.671

**Table 4:** Percentiles at birth and at 28 days of life, by group according to growth velocity.

	Total at Birth	Total at 28 Days of Life	Group 1 at 28 Days of Life	Group 2 at 28 Days of Life
Weight	32.7 ± 23.1	8.5 ± 12.2	4.2 ± 6.2	11.7 ± 14.6
Size	31.2 ± 26.1	12.4 ± 17.2	10.3 ± 15.2	13.9 ± 18.8
HC	38.0 ± 29.0	13.4 ± 21.1	13 ± 21.7	13.7 ± 21.3

HC: Head circumference.

## Data Availability

All data collected is available upon request to the corresponding author.

## Abbreviations

The following abbreviations are used in this manuscript:

GV	Growth Velocity
HC	Head Circumference
PNB	Preterm Newborn(s)
NICU	Neonatal Intensive Care Unit
HRAEV	Hospital Regional de Alta Especialidad Victoria
WHO	World Health Organization
GA	Gestational Age
NB	Newborn
AAP	American Academy of Pediatrics
ELBW	Extremely Low Birth Weight
IVH	Intraventricular Hemorrhage
IUGR	Intrauterine Growth Restriction
RNP	Recién Nacido Pretérmino (Preterm Newborn—Spanish abbreviation)
LBW	Low Birth Weight
NEC	Necrotizing Enterocolitis
RDS	Respiratory Distress Syndrome
DAP	Ductus Arteriosus Persistente (Patent Ductus Arteriosus—PDA)
PN	Parenteral Nutrition
SDG	Semanas de Gestación (Weeks of Gestation—Spanish abbreviation)
SPSS	Statistical Package for the Social Sciences
IGF	Insulin-like Growth Factor
IGFBP-3	Insulin-like Growth Factor Binding Protein 3
CI	Confidence Interval

## References

[1] Blencowe H., Cousens S., Chou D., Oestergaard M., Say L., Moller A.B., Kinney M., Lawn J. (2013). Born Too Soon: The Global Epidemiology of 15 Million Preterm Births. Reprod. Health.

[2] Beck S., Wojdyla D., Say L., Betran A.P., Merialdi M., Requejo J.H., Rubens C., Menon R., Van Look P.F.A. (2010). The Worldwide Incidence of Preterm Birth: A Systematic Review of Maternal Mortality and Morbidity. Bull. World Health Organ..

[3] Hoffman H.J., Stark C.R., Lundin F.E., Ashbrook J.D. (1974). Analysis of Birth Weight, Gestational Age, and Fetal Viability, U.S. Births, 1968. Obstet. Gynecol. Surv..

[4] Zamorano C., Guzmán J., Baptista H., Fernández L. (2012). Pérdida de Peso Corporal y Velocidad de Crecimiento Postnatal En Recién Nacidos Menores de 1,500 Gramos Durante Su Estancia En Un Hospital de Tercer Nivel de Atención. Perinatol. Reprod. Hum..

[5] Martin C.R., Brown Y.F., Ehrenkranz R.A., O’Shea T.M., Allred E.N., Belfort M.B., McCormick M.C., Leviton A. (2009). Nutritional Practices and Growth Velocity in the First Month of Life in Extremely Premature Infants. Pediatrics.

[6] Ehrenkranz R.A., Dusick A.M., Vohr B.R., Wright L.L., Wrage L.A., Poole W.K. (2006). Growth in the Neonatal Intensive Care Unit Influences Neurodevelopmental and Growth Outcomes of Extremely Low Birth Weight Infants. Pediatrics.

[7] Ehrenkranz R.A., Younes N., Lemons J.A., Fanaroff A.A., Donovan E.F., Wright L.L., Katsikiotis V., Tyson J.E., Oh W., Shankaran S. (1999). Longitudinal Growth of Hospitalized Very Low Birth Weight Infants. Pediatrics.

[8] Fenton T.R., Kim J.H. (2013). A Systematic Review and Meta-Analysis to Revise the Fenton Growth Chart for Preterm Infants. BMC Pediatr..

[9] Fenton T.R. (2003). A New Growth Chart for Preterm Babies: Babson and Benda’s Chart Updated with Recent Data and a New Format. BMC Pediatr..

[10] Hojsak I., Chourdakis M., Gerasimidis K., Hulst J., Huysentruyt K., Moreno-Villares J.M., Joosten K. (2021). What Are the New Guidelines and Position Papers in Pediatric Nutrition: A 2015–2020 Overview. Clin. Nutr. ESPEN.

[11] Lango M.O., Horn A.R., Harrison M.C. (2013). Growth Velocity of Extremely Low Birth Weight Preterms at a Tertiary Neonatal Unit in South Africa. J. Trop. Pediatr..

[12] Hair A.B., Hawthorne K.M., Chetta K.E., Abrams S.A. (2013). Human Milk Feeding Supports Adequate Growth in Infants ≤ 1250 Grams Birth Weight. BMC Res. Notes.

[13] Espinosa-Reyes T.A., Ladrón-de-Guevara-Casals A., Carvajal-Martínez F., Domínguez Alonso E. (2013). Natal Growth of Very Low Birthweight Pre-Term Neonates. Rev. Cubana Endocrinol..

[14] García-Muñoz R.F., Figueras Aloy J., Saavedra Santana P., García-Alix A. (2017). Postnatal Growth at Hospital Discharge in Extremely Premature Newborns in Spain. An. Pediatr..

[15] Raturi S., Zheng Q., Daniel L.M., Shi L., Rajadurai V.S., Agarwal P.K. (2017). Nutritional Intake and Growth Velocity in Preterm Extremely Low-Birthweight Infants in Asia: Are We Doing Enough?. J. Paediatr. Child. Health.

[16] Khan Z., Morris N., Unterrainer H., Haiden N., Holasek S.J., Urlesberger B. (2018). Effect of Standardized Feeding Protocol on Nutrient Supply and Postnatal Growth of Preterm Infants: A Prospective Study. J. Neonatal Perinatal Med..

[17] Avilés L.M., Morán R.J.G., Méndez A.M.R., Leboreiro J.I., Zapata I.B., Bronstein A.B. (2018). Evaluación Del Patrón de Crecimiento Postnatal y Factores Asociados En Neonatos Pretérmino. An. Médicos De. La. Asoc. Médica Del. Cent. Médico ABC.

[18] Acevedo-Olguín A.L., Iglesias-Leboreiro J., Bernárdez-Zapata I., González-Morán R.J., Rendón-Macías M.E. (2018). Crecimiento Ponderal Intrahospitalario En Pretérminos de Peso Adecuado y Bajo al Nacimiento. Rev. Mex. Pediatr..

[19] Marcus C.L., Carroll J.L., Koerner C.B., Hamer A., Lutz J., Loughlin G.M. (1994). Determinants of Growth in Children with the Obstructive Sleep Apnea Syndrome. J. Pediatr..

[20] Nieminen P., Löppönen T., Tolonen U., Lanning P., Knip M., Löppönen H. (2002). Growth and Biochemical Markers of Growth in Children with Snoring and Obstructive Sleep Apnea. Pediatrics.

